# Self-reported cognitive and affective complaints associated with olfactory loss in an online survey of individuals with COVID-19

**DOI:** 10.1007/s00405-025-09660-x

**Published:** 2025-10-18

**Authors:** Christophe Bousquet, Veronica Pereda-Loth, Denis Pierron, Mauricio Gonzalez-Navarro, Santiago Avila-Ríos, Nathalie Mandairon, Camille Ferdenzi, Moustafa Bensafi

**Affiliations:** 1 Lyon Neuroscience Research Center, CNRS UMR5292, INSERM U1028, Université Claude Bernard Lyon 1, Bron, France; 2 Department of Psychology, University of Konstanz, Konstanz, Germany; 3 Centre for the Advanced Study of Collective Behaviour, University of Konstanz, Konstanz, Germany; 4 Équipe de Médecine Évolutive, Faculté de chirurgie dentaire, URU- Evolsan, Université de Toulouse, Toulouse, France; 5 Faculté de médecine Rangueil, GSBMS, Université de Toulouse, Toulouse, France; 6 Otolaryngology subdivision, National Institute of Rehabilitation Luis Guillermo Ibarra Ibarra, Mexico City, Mexico; 7 Centre for Research in Infectious Diseases, National Institute of Respiratory Diseases, Mexico City, Mexico

**Keywords:** Anosmia, Cognition, Mood, long-COVID, Symptoms, Olfaction

## Abstract

**Purpose:**

The symptomatology associated with COVID-19 is very diverse, ranging from flu-like symptoms to those affecting olfaction, cognition or mood for long periods. The present study explored the associations between self-reported olfactory deficits and cognitive and emotional complaints in a large-scale online survey conducted among individuals who had COVID-19.

**Methods:**

Two complementary online studies were set up, one in France and the other in Mexico, involving 3108 and 364 volunteers respectively, to investigate the link between olfactory loss in COVID-19 and self-reported cognitive and emotional changes. Cognitive and affective complaints were assessed using simple yes/no items inspired by previously published studies, but not based on standardized clinical questionnaires.

**Results:**

A first result was that cognitive difficulties are more frequent in COVID-19 individuals with long-standing olfactory disorders than in patients who have recently developed olfactory disorders. In addition, we also showed that the prevalence of cognitive difficulties is higher in COVID-19 patients with olfactory disorders than in those without. Furthermore, cognitive difficulties in patients with long-term olfactory disorders are more strongly associated with memory difficulties than with attention difficulties. Finally, mood disorders were more frequent in COVID-19 participants with olfactory loss than in those without.

**Conclusion:**

Taken together, these data suggest that in COVID-19, the duration of olfactory loss is a key factor, strongly associated with cognitive and affective impairment. These data should provide us with further guidance on the management of people affected, which should not simply be unimodal, targeting just one category of symptoms.

**Supplementary Information:**

The online version contains supplementary material available at 10.1007/s00405-025-09660-x.

## Introduction

COVID-19 is associated with a specific symptomatology that can combine a flu-like condition, respiratory disorders and loss of taste and smell without nasal obstruction [1]. Smell deficits (or Olfactory Disorder, OD) have been shown to affect an average of 40% of people positive to COVID-19, with strong regional variations [2]. In addition to these geographical variations, differences in prevalence of OD between variants of the SARS-CoV-2 can be very large: the Alpha and Delta variants have very high prevalence of OD (between 30% and 50% on average), whereas the original SARS-CoV-2 and the Omicron variant only have a prevalence of OD around 10% [3]. These large differences could be explained by different infection processes between variants, with Alpha and Delta variants more likely to enter and damage support cells of the olfactory neurons, leading to higher levels of OD [4].

In all cases, although a majority of patients recover from these losses within a few days or weeks [5, 6], many show OD that may still persist more than 6 months after infection [6–9]. These long-term smell disorders in COVID-19 patients are included in the clinical picture of so-called long-COVID. In addition to these ODs, long-COVID is also characterised by cognitive difficulties affecting memory processes [10–13] and sometimes attention and concentration [10, 11, 14]. Depression and fatigue are also very commonly reported by patients suffering from long-COVID-associated ODs [11]. Although long-term olfactory losses on the one hand and cognitive and emotional impairments on the other are increasingly well documented in COVID-19, it is not yet clear what the relationship is between the two forms of deficits. Are cognitive, emotional and/or memory difficulties more frequent in COVID-19 individuals who suffer from long OD than in those who do not? This article will attempt to provide some descriptive elements of answers to this question. The interest in this issue is obvious when we want to better understand the symptomatology of long-COVID, but also to provide elements of knowledge that shed new light on the effects of olfactory losses on quality of life and cognition, well documented even before the COVID crisis [15, 16].

To do so, we conducted an online study involving more than 3,100 French volunteers positive for COVID-19 and who reported an olfactory loss, thus covering the first 3 major epidemic waves that affected France. Participants were asked to complete a questionnaire on their memory and attentional abilities during their loss of smell inspired by a previous study showing that the level of cognitive difficulties (attention and memory) of patients can be assessed using basic binary questions (yes/no) [17]. In particular, they were asked to report whether or not, in addition to their OD, they had difficulty to: i/ remember events, dates, appointments, ii/ remember the location of objects, iii/ remember old memories, iv/ remember very recent activities, v/ follow a program on television or radio, vi/ follow a conversation that was intended for them. Respectively, the 6 items were selected to cover different aspects of attention and memory: temporal, spatial, long-term, short-term, concentration and working memory. With this survey, we investigated whether a specific type of cognitive difficulty was more frequent in COVID-19 participants reporting an OD and whether cognitive difficulties were more frequently reported as the OD lasted longer. As over the course of the study the main circulating variant of the SARS-CoV-2 virus varied, we also assessed whether the Alpha variant was associated with more or less cognitive difficulties compared to the Beta or the Gamma variants.

To extend the results of the French population, we took advantage of a large-scale study on the determinants of COVID-19 infection in Mexico. From this Mexican online survey, we could estimate the extent of the relationship between cognitive difficulties and loss of smell (as in the French sample), but also to understand how the mood and anxiety of those affected were related to cognitive difficulties and olfactory loss. Such a relationship exists in other long term medical conditions and may prevent medical recovery [18]. For example, 6 months after a stroke, participants suffering from cognitive impairment were more likely to express depressive symptoms rather than anxiety symptoms [19].

## Results

### The probability to develop a cognitive difficulty increases with the duration of olfactory disorders– French survey

**Fig. 1:**
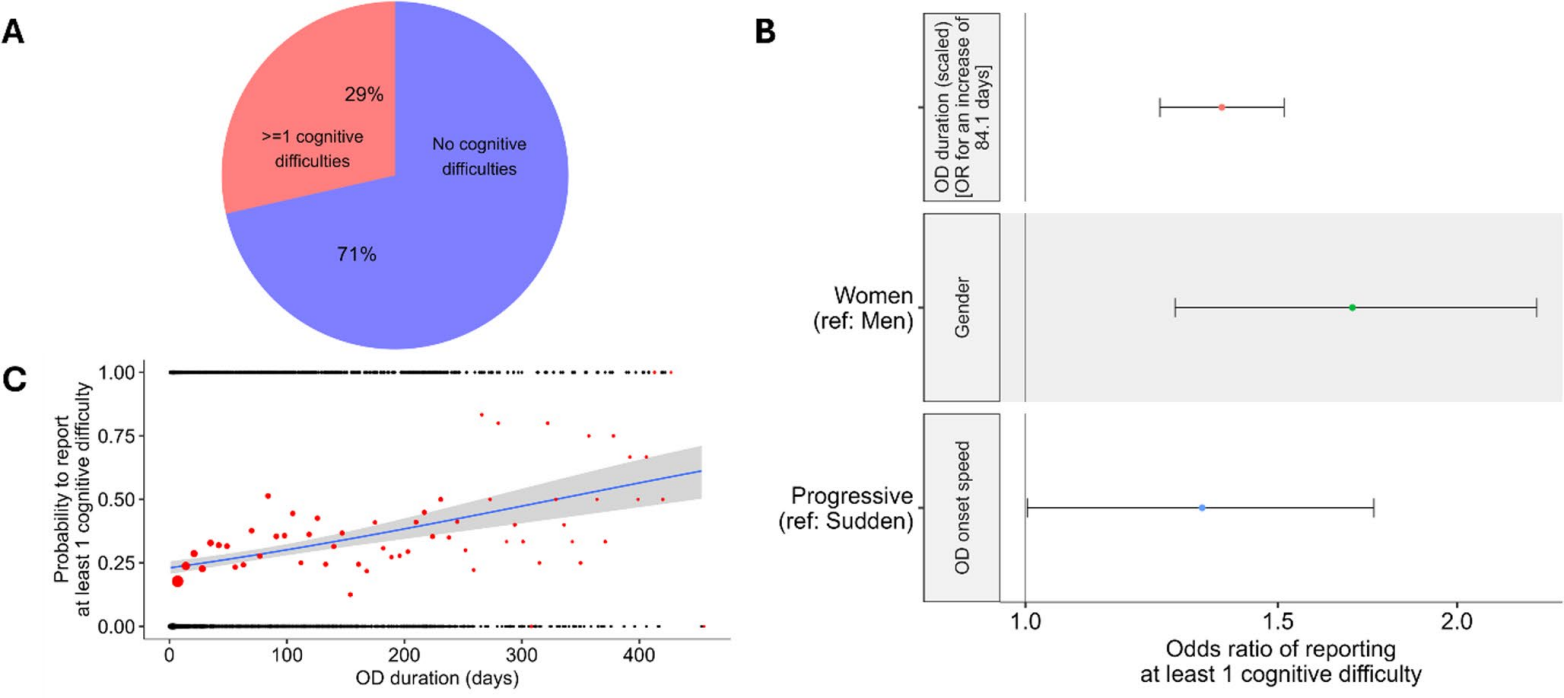
(**A**) Pie chart representing the percentage of participants reporting at least 1 cognitive difficulty (29%, red portion) or no cognitive difficulty (71%, blue portion). The other panels illustrate the significant variables affecting the probability to report at least 1 cognitive difficulty. (**B**) Odds-ratios (OR) and 99% confidence intervals of the significant variables. Note that for continuous variables, OR are given for each standard deviation of the corresponding variable. (**C**) Effect of OD duration. In C, the black points represent raw data, while for illustrative purposes the red points represent the probability for participants binned per week; the size of black and red points vary according to their sample size

**Fig. 2:**
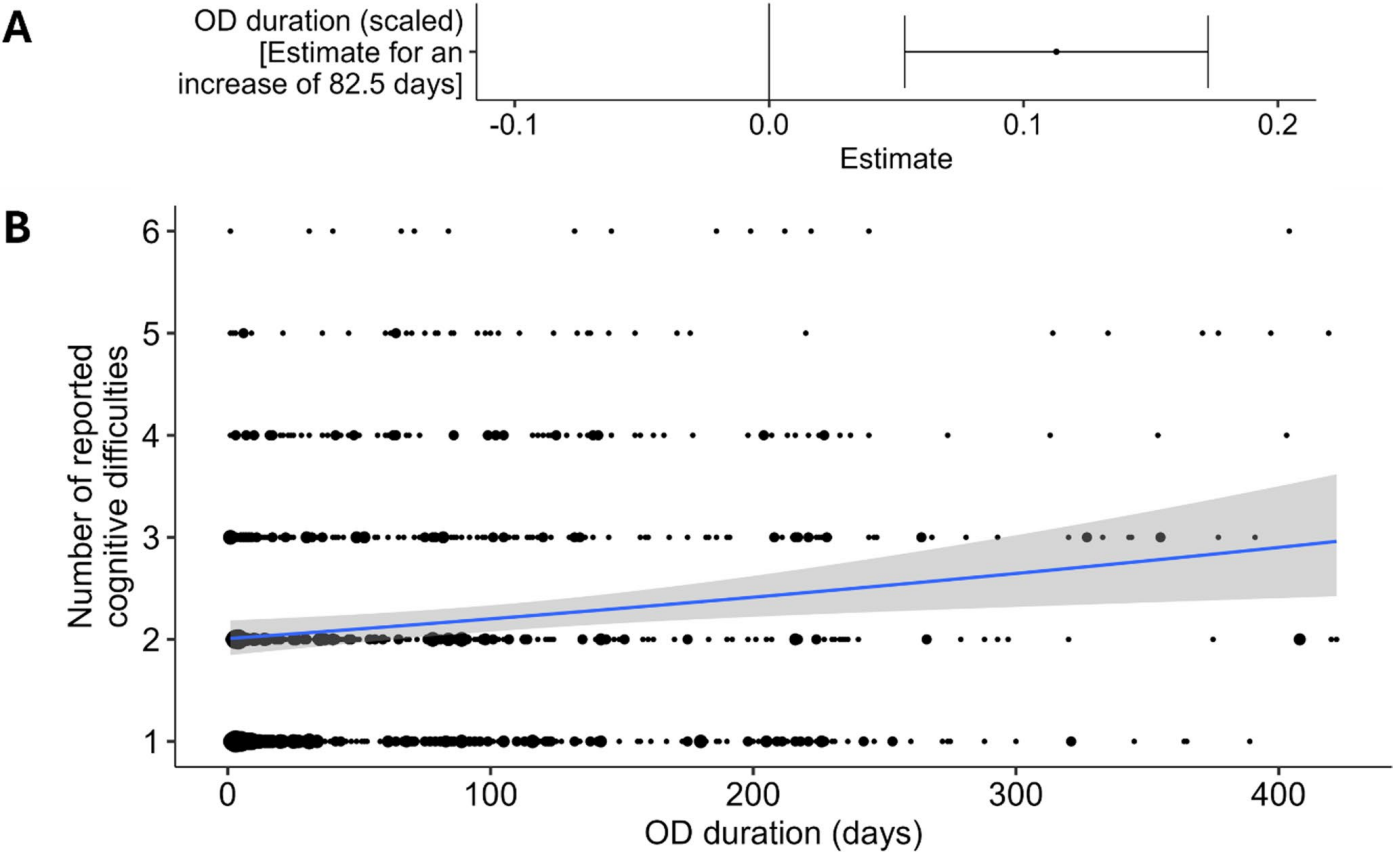
Significant variables related to the number of reported cognitive difficulties. (**A**) Model estimate (for 1 standard deviation) and (**B**) Effects of OD duration on the number of reported cognitive difficulties. In B, circle size is proportional to the number of participants, the blue line and the grey shaded area display the prediction from the model and its associated 95% confidence interval, respectively

A first descriptive analysis of the French survey showed that around 29% of participants with COVID-19-related OD reported at least 1 cognitive difficulty (Fig. 1A). The hurdle model used (see Methods) enabled us to determine first which factors influence the probability to report at least 1 cognitive difficulty and then, which factors influence the number of reported cognitive difficulties. The probability to report at least 1 cognitive difficulty: i/ increased consistently with OD duration (logistic regression part of the minimal hurdle model: β = 0.32 ± 0.04, z = 8.17, *p* < 0.001, OR = 1.41 [1.29–1.54]) (Fig. 1B and C), ii/ was significantly higher in women (29.8%) than in men (20.5%) (β = 0.53 ± 0.11, z = 4.66, *p* < 0.001, OR = 1.61 [1.24–2.11]) (Fig. 1B) and iii/ was higher when OD appeared progressively (31.2%) rather than suddenly (27.4%) (β = 0.28 ± 0.11, z = 2.63, *p* < 0.01, OR = 1.30 [1.01–1.68]) (Fig. 1B). Fig. S1 illustrates the evolution of the frequency to report at least 1 cognitive difficulty as a function of OD duration. No other variable explained the probability to report at least 1 cognitive difficulty. Concerning the number of reported cognitive difficulties, only OD duration had a significant effect, with longer OD associated with larger numbers of cognitive difficulties (zero-truncated Poisson regression part of the minimal hurdle model: β = 0.12 ± 0.02, z = 5.71, *p* < 0.001) (Fig. 2). No other variable explained the number of reported cognitive difficulties.

### The effect of long OD duration is present for nearly all types of cognitive difficulty – French survey

**Fig. 3:**
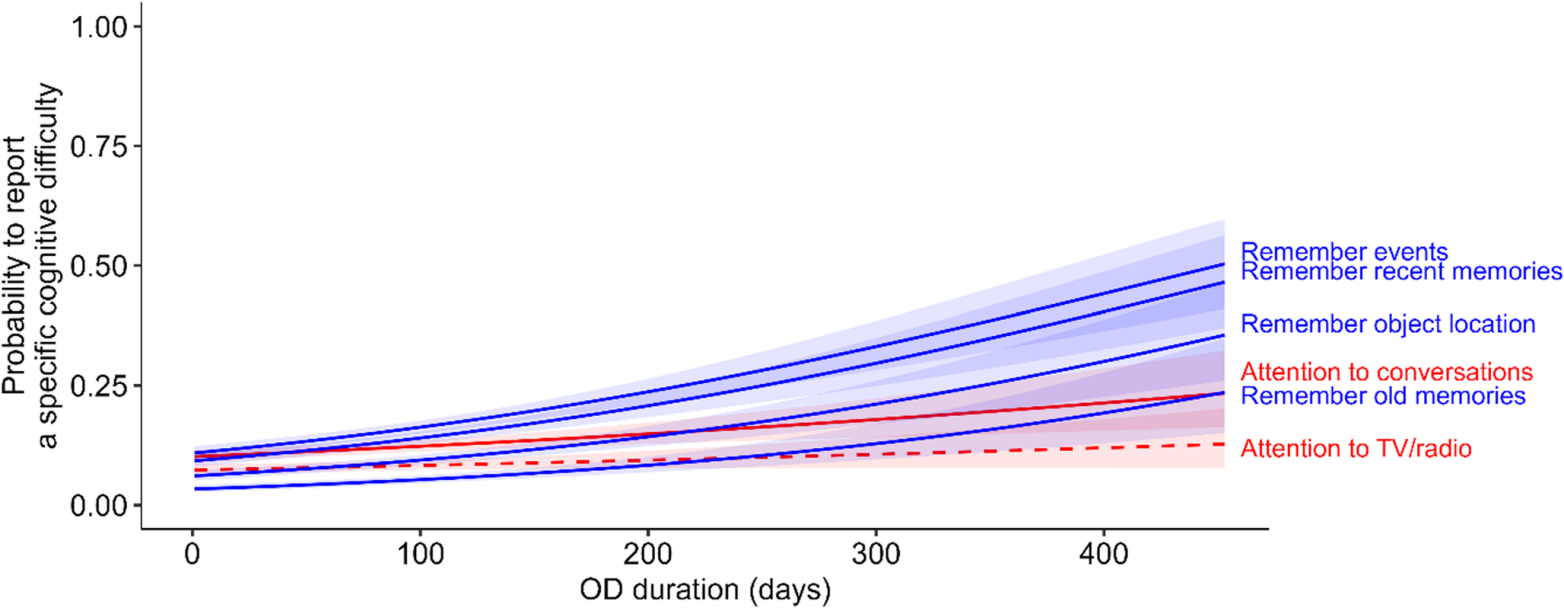
Blue and red lines correspond to items dealing with memories and with attention, respectively. Plain and dashed lines correspond, respectively, to a significant and a non-significant effect of OD duration on the probability (estimated from a logistic regression) to report a cognitive difficulty. Shaded areas around the lines represent their associated 95% confidence intervals

One question raised by the above findings is whether this association between duration of OD and cognition is observed for all types of questionnaire items analysed (e.g., memory, attention) or only for a subcategory of them. To test this, we ran 6 logistic regressions (1 for each item) with the presence or absence of the corresponding questionnaire item as the response variable and the OD duration as the explanatory variable. Among the 6 questionnaire items, 5 showed a significant relationship with OD duration (Remember events: β = 0.39 ± 0.04, z = 8.92, *p* < 2e-16, OR = 1.48 [1.32–1.66]; Remember recent memories: β = 0.40 ± 0.05, z = 8.65, *p* < 2e-16, OR = 1.49 [1.32–1.68]; Remember object location: β = 0.40 ± 0.05, z = 7.52, *p* = 5.68e-14, OR = 1.49 [1.30–1.70]; Remember old memories: β = 0.41 ± 0.07, z = 6.13, *p* = 8.8e-10, OR = 1.50 [1.26–1.77]; Attention to conversations: β = 0.18 ± 0.05, z = 3.64, *p* = 0.0003, OR = 1.20 [1.05–1.37]) and one did not (Attention to TV/radio: β = 0.11 ± 0.06, z = 1.85, *p* = 0.06, OR = 1.12 [0.95–1.31]) (Fig. 3). Note that the questionnaire items dealing with memory difficulties were those with the highest odd-ratios.

### The type of SARS-CoV-2 variant does not influence the relationship between OD duration and cognitive difficulties – French survey

**Fig. 4:**
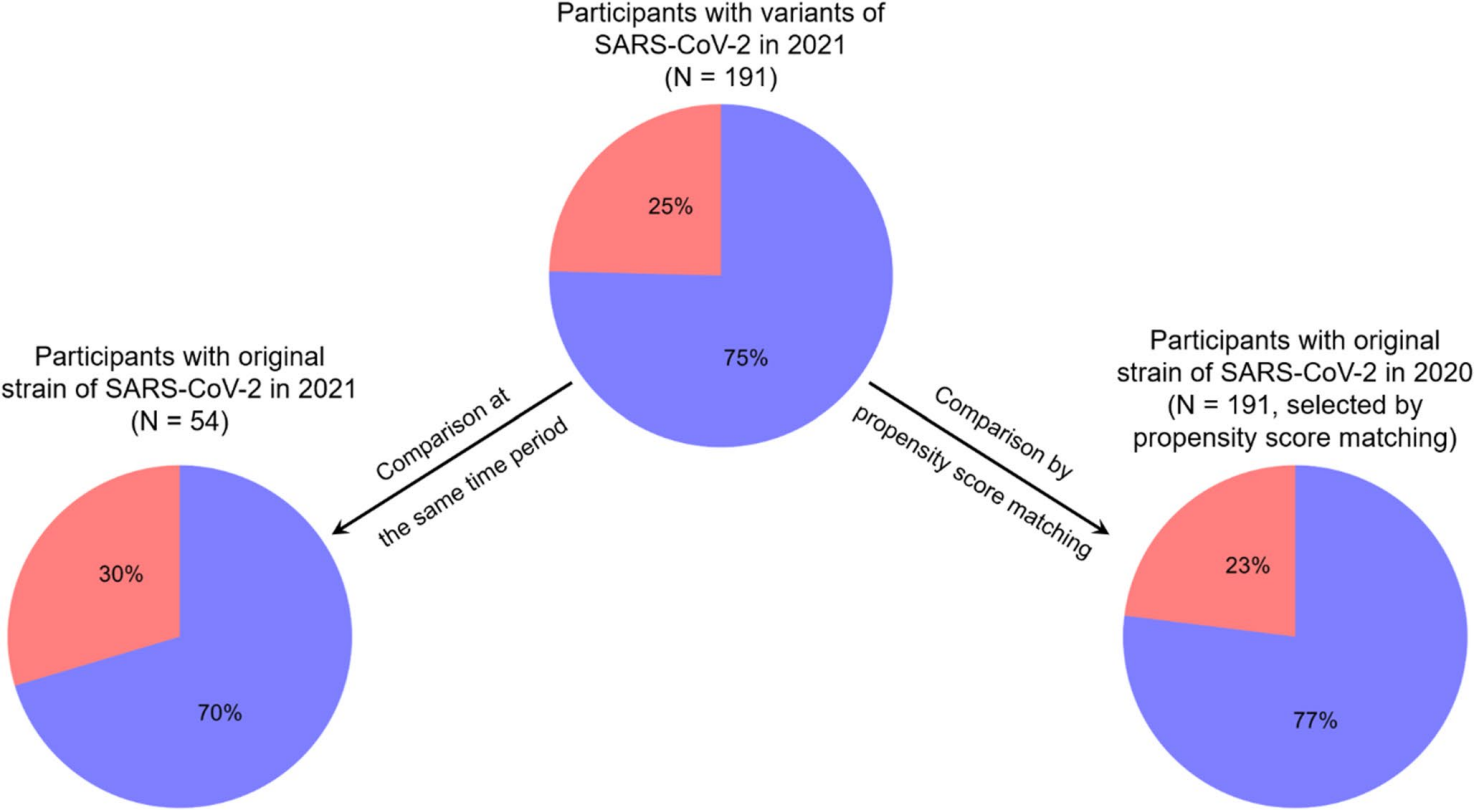
Pie chart presenting the proportion of participants who reported at least 1 cognitive difficulty (red portion) or no cognitive difficulty (blue portion) as a function of virus strains. In the top chart, participants had one of the variants of SARS-CoV-2 with associated OD. In the chart on the left, participants had the original strain of SARS-CoV-2 and at the same time period than for the top chart but were much rarer. In the chart on the right, participants had the original strain of the SARS-CoV-2 with associated OD. They were matched by propensity score matching to the characteristics (according to age, gender, OD type, OD onset speed, OD constancy, OD duration, month of COVID-19 onset, smoking status and BMI) of the participants in the top chart

As the SARS-CoV-2 virus has mutated since the beginning of the pandemic, we examined whether this association between OD and cognitive difficulties was comparable and similar in individuals having been infected with the original strain of the virus compared to individuals having been infected with other variants of the virus (e.g., so-called Alpha variant). In our sample, the vast majority of variants were of the Alpha type (88.0%), followed by the Beta or Gamma type (8.9%) and by variants for which the type is not known (3.1%). To do this, we compared the effect of SARS-CoV-2 variant on OD-related cognitive difficulties in two ways. With a χ^2^-test, we compared the proportion of participants infected by a variant and who reported at least 1 cognitive difficulty to the proportion of participants infected by the original SARS-CoV-2 in the same time window and who reported at least 1 cognitive difficulty. We chose to perform a binary analysis comparing individuals without vs. with (at least one) cognitive deficit since it is likely that the appearance of the first cognitive deficit is the most detrimental to daily life, compared to additional deficits. Both groups experienced COVID-19 in the same period and are thus temporally directly comparable. Results showed that the infection with a variant of the SARS-CoV-2 did not affect the proportion of participants reporting at least 1 cognitive difficulty. This proportion was similar between participants infected by a variant in 2021 and participants infected by the original virus also in 2021 (24.6% vs. 29.6%, respectively; χ^2^ = 0.32, df = 1, *p* = 0.57). Since variants by definition appeared later than the original SARS-CoV-2, when comparing the same time window, we have an imbalance between the number of participants infected by a variant (191 participants) and the number of participants infected by the original coronavirus (54 participants), which could potentially bias the results. Therefore, in a second step, we selected 191 participants infected by the original virus in 2020, matched with those infected by a variant for potentially confounding covariates (see Methods). Again, the comparison between the participants infected by a variant and their matched participants was not significant (24.6% vs. 23.0%, respectively; χ^2^ = 0.06, df = 1, *p* = 0.81) (Fig. 4).

### Cognitive difficulties in Covid-19 patients appear more frequent with than without OD – Mexican survey

Another question raised by our findings is whether this average prevalence of 29% of cognitive difficulties in COVID-19 positive individuals is specific to those with an OD? To answer this question, we used the survey carried out in Mexico in which we compared the prevalence of at least one cognitive difficulty in COVID-19 patients with and without OD. The first result of interest of this online survey was that 56.6% of patients positive to COVID-19 (206 out of 364) declared an OD, a prevalence comparable to that observed in France (60.9%; 389 out of 639) [20, 21]. Moreover, the prevalence of cognitive difficulties in participants with OD was 26.7% [55 out of 206 participants, very close to the estimate in France] and was significantly higher than the prevalence of cognitive difficulties in participants without OD [17.7%, 28 out of 158 participants] (one-sided χ^2^ = 3.60, df = 1, *p* = 0.029).

### The co-occurrence of cognitive difficulties and mood disorders is greater with than without OD – Mexican survey

**Fig. 5:**
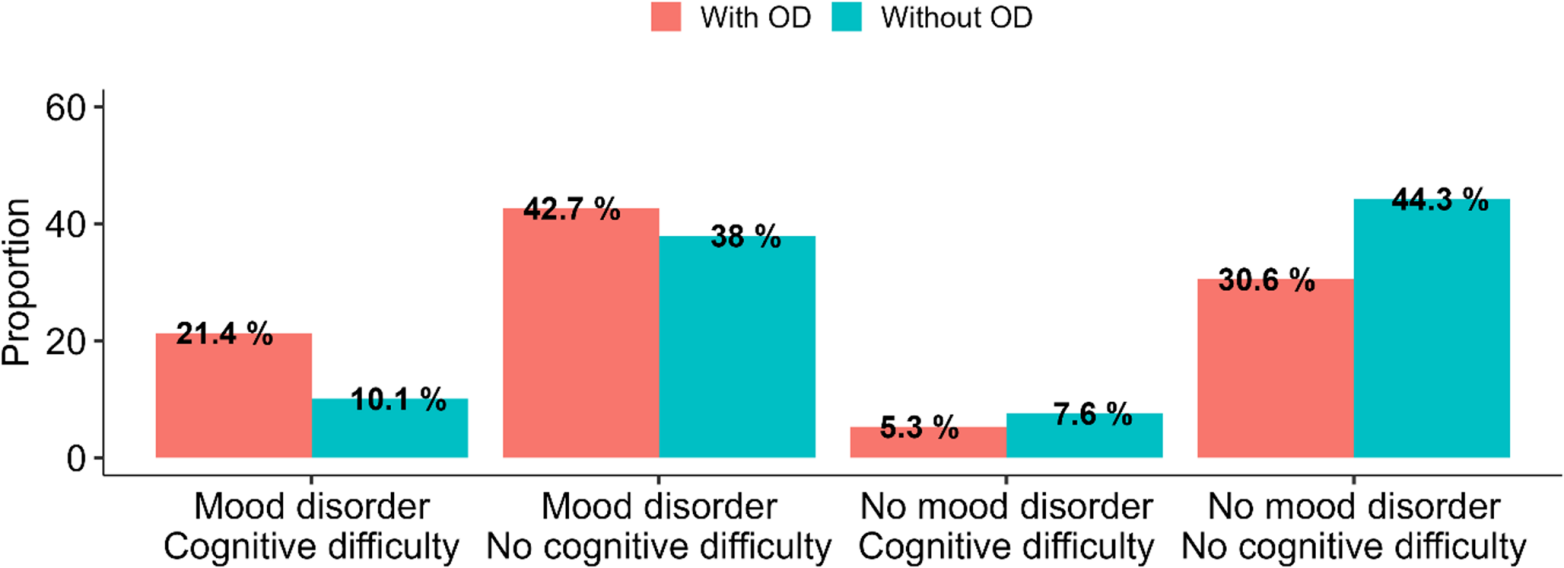
Effect of the presence of OD (left, pink bars: with OD; right, blue bars: without OD) on the simultaneous presence of either mood disorder or cognitive difficulty

Given the existing relationship between OD and mood disorders (e.g., anxiety, depression), we further asked the question of whether mood changes could also be involved in this relationship between OD and cognitive difficulties in this patient population. We found that reporting at least one mood disorder was more frequent in COVID-19 participants with OD (64.1%, 132 participants out of 206) than in COVID-19 participants without OD (48.1%, 76 out 158 participants). This difference was statistically significant (χ^2^ = 8.68, df = 1, *p* = 0.003). Furthermore, the combined presence of mood disorders and cognitive difficulties was more frequent for participants with OD (21.4%, 44 participants out of 206) than for participants without OD (10.1%, 16 participants out of 158) (logistic regression, χ^2^ = 8.56, df = 1, *p* < 0.01; Fig. 5).

## Discussion

The main aim of the present study was to characterize the prevalence of cognitive and affective difficulties in people with COVID-19-related olfactory loss. A first finding of interest is that, within the time frame of the study, cognitive difficulties were more frequent in COVID-19 individuals with longer OD duration. Moreover, we also showed that the prevalence of cognitive difficulties is higher in COVID-19 patients with OD compared to those without OD. There are different potential explanations for these complementary findings. One hypothesis concerns a possible infection of the brain by the virus, leading to inflammation and neuronal damage. Clinical studies in humans have demonstrated oedema of the olfactory bulb in affected individuals, without however demonstrating the presence of the virus in the human brain [22]. Furthermore, a study carried out on hamsters detected the presence of SARS-CoV-2 not only in the olfactory bulb, but also in areas of the brain further away from the cerebral cortex and brain stem [23], a viral presence that may lead to neurological symptoms and cognitive and olfactory difficulties. Here, long cognitive and olfactory alterations would not necessarily be the consequence of each other but would have a common neuronal cause. A second hypothesis may imply a causal relationship. On the one hand, alterations in quality of life induced by olfactory loss [16, 20, 24, 25] could have an impact on the person’s affective state and stress, leading to impaired cognitive functions. On the other hand, impairment of cognitive functions is in turn associated to odour identification deficits in aged individuals [26].

The present study does not allow us to decide between these different hypotheses, but whatever the cause, it suggests that gender, but not age, has an impact on this relationship between OD and cognitive difficulties. The absence of any effect of age may seem surprising, given its known association with the duration of OD [24]. In our case, statistical analysis revealed a stronger effect of the disorder duration factor than of the age factor, which was dropped during the model selection process. The cause of the gender effect is still unclear, and further studies are needed to better understand the underlying socio-cognitive and/or biological mechanisms. One possibility is that women are more likely to report and describe their symptoms [24]. Another possibility would be linked to a different physiological or hormonal influence between men and women [27].

On a different front, we showed no influence of the type of COVID-19 variants on this relationship between duration of OD and cognitive difficulties. This association may be more broadly related to infection with the virus itself, rather than to specific characteristics of the variants. It is to be noted that our dataset does not include patients who suffered from the Omicron variant and other later variants. It is therefore not sure if the Omicron variant would display a different relationship between OD duration and cognitive difficulties, particularly because Omicron induces less OD in the first place [3]. Besides, as for any questionnaire study, we did not control the veracity of the variant reported by the participants, but we trusted the result they reported from their PCR test.

Another finding of interest is that effects on cognitive complaints were more pronounced for certain questionnaire items than others, especially for those related to memory. This result raises the question of the relationship between smell and memory, on the one hand, and smell and attentional processes, on the other. While it has been widely documented that olfactory networks integrate memory areas such as the entorhinal cortex or hippocampus [28], the overlap between smell and attention has been little documented [29, 30] and deserves to be better characterized in order to understand the mechanisms underlying our pattern of results.

A final finding of interest concerns mood disorders, which are more frequent in COVID-19 participants with OD than in those without. Furthermore, the presence of mood disorders was more associated with cognitive impairment in participants with OD. These data suggest complex links between these chemosensory, affective and cognitive symptoms. The sense of smell is closely linked to the limbic system [28], a neural network that is also involved in emotions. It cannot therefore be ruled out that OD may lead to an alteration in this connection between the olfactory system and emotion regulation, thus contributing to a higher prevalence of mood disorders in people reporting an OD. In turn, these mood disorders may contribute to the impairment of certain cognitive functions by, as mentioned above, generating unusual levels of stress and anxiety. In the case of people without OD, the lack of association between mood and cognitive alterations suggests that the cognitive difficulties observed in these people may be influenced by other factors, such as the severity of COVID-19 infection, comorbidities or other specific mechanisms not directly linked with olfaction.

This study has several limitations. First, the data collected are based exclusively on self-assessments, which may introduce subjective biases, particularly when estimating cognitive, emotional, and sensory impairments. Second, the absence of objective measures—such as standardized neuropsychological tests or clinical mood assessments—limits the external validity of the results. Third, the cross-sectional nature of the study does not allow for the establishment of causal relationships: the observed disorders could be induced by, an indirect consequence of, or a manifestation of pre-existing vulnerabilities to COVID-19. In particular, as pre-infection data are unavailable, it is impossible to ascertain whether memory and mood disorders were present prior to the disease. Fourth, selection bias remains a possibility as individuals with more prominent or persistent symptoms may be more inclined to participate in an online study, which limits the generalisability of the results. This may have led to an overrepresentation of participants with more severe or prolonged symptomatology. As a result, our findings may not fully reflect the broader population of individuals who experienced COVID-19 without major complaints. However, we believe that the patients who decided to take part in our surveys are also the ones most likely to book consultations at the clinic. To complement our approach, longitudinal studies are needed to better understand how symptoms evolve over time, and to determine whether they are caused by, or simply revealed by, the infection. It would also be important to include standardised clinical assessments, as well as pre- and post-infection data where possible. Finally, exploring the underlying neurobiological mechanisms, including links between olfactory disorders and cognitive or emotional impairments, would significantly advance our understanding of the neurological effects of COVID-19.

In conclusion, despite its qualitative approach via questionnaires, the present study has highlighted several results linking olfactory loss, cognitive and affective alterations in COVID-19. The duration of OD is a key factor, strongly associated with cognitive and affective alterations. This shed light on the management of those affected, which must not be merely unimodal by targeting just one category of symptoms. Management of long-COVID must take into account all the symptoms of COVID-19, starting with sensory and cognitive loss via medical care, but also taking into account psychological and socio-professional care [20] in order to reduce the impact of emotional problems.

## Methods

### French online survey

#### The participants

The French online participative study was advertised at the national level in France and intended for people with OD, be it caused by COVID-19 or not. In this article, we included participants who (i) have not yet recovered from their OD and (ii) were diagnosed COVID-19 positive by a health practitioner and based on a laboratory test (75.2%: PCR-test after nasal swab, 0.3%: chest radio 10.0%: other [e.g., blood test 9.7%, unknown 0.3%]) or based on their symptoms (14.5%). Although the diagnosis based only on the symptoms has a higher rate of uncertainty, we used it as an inclusion criterion because at the beginning of the pandemic many patients were diagnosed this way due to very limited access to PCR tests. Other inclusion criteria were: (iii) responding to the full questionnaire (i.e., no dropout before the end of the questionnaire), (iv) completing the questionnaire for the first time, (v) aged between 18 and 99 years old, (vi) answering between 08/04/2020 until 19/05/2021, and (vii) providing usable data on OD duration and declaring OD onset maximum 1 week prior to COVID-19 onset. In total, responses of 3108 individuals were included. Participants were mostly women (81.1%) and were aged (Mean ± SD) 40.8 ± 12.4 years (range 18–81). Men and women did not differ on age (40.7 ± 13.0 and 40.8 ± 12.3 years respectively; t_3108_ = 0.122, *p* = 0.902). All inclusion criteria are described in Fig. S2 and the characteristics of the participants after the exclusion criteria are presented in Table 1.

**Table 1:** Characteristics of the participants who took part in the French and the Mexican online surveys

Survey	French	Mexican
Participants before exclusion criteria	12 123	3 051
Participants after exclusion criteria	3 696	364
- Participants with OD	3 696 (100%)	206 (56.59%)
- Participants without OD	0 (0%)	158 (43.41%)
Age in years (m ± sd)	40.8 ± 12.4	39.1 ± 10.3
Sex assigned at birth		
- Female	2 521 (81.11%)	228 (62.64%)
- Male	587 (18.89%)	133 (36.54%)
- Missing	0 (0%)	3 (0.82%)

The full description of the exclusion criteria can be found in Fig. S2 and S3 for the French and the Mexican online survey, respectively

#### The questionnaire

The online questionnaire was accessible from a French information website dedicated to the sense of smell (https://project.crnl.fr/odorat-info/). It was advertised at the national level in France through different channels (numerous audio-visual media communications, authors’ professional and personal networks, advertisements posted in local health centres and pharmacies). The call was directed to people who noticed a change in their sense of smell, be it caused by COVID-19 or not, and be it still present or not. The stated purpose of the study was to help us better understand how OD manifest themselves and what impact they have on quality of life, especially in the context of the COVID-19 crisis. The questionnaire comprised a series of sections including among others demographic information, COVID-19 related information and a questionnaire on cognitive difficulties (see Supplementary Note 1). The latter questionnaire was as follows: “At the time (and/or after) these changes in smell and/or taste occurred, you also had difficulty: 1/ To remember events, dates, appointments, 2/ To remember the location of objects, 3/ To remember old memories, 4/ To remember very recent activities, 5/ To follow a program on television or radio, 6/ To follow a conversation that was intended for you, 7/ None of the above”.

The questionnaire was completed only once by the participants (i.e., no follow-up study was planned), providing information at a single time-point. Participants responded at varied times after the onset of the disease (and after the onset of their COVID-19 related OD).

### Mexican online survey

#### The participants

Unlike the French questionnaire, the Mexican online survey targeted the general population, with and without COVID-19. In total, 3051 participants completed the survey, of which 364 had COVID-19 (11.9%) and are the focus of our investigation. The Mexican data are derived from a large-scale epidemiological study conducted using an online questionnaire over a 6-month period, targeting individuals with positive COVID-19 diagnoses, suspected cases, contacts of confirmed cases, and those experiencing symptoms during the contingency. Promotion was carried out through social media and other communication channels. Unlike the French study, no information on the type of symptoms required (e.g., olfaction for the French study) was included in the call for participation, in order to have access to information on the prevalence of COVID symptoms without introducing selection bias. The study was carried out by an international multidisciplinary collaboration comprising, specialists at INER (National Institute of Respiratory Diseases, Mexico City), as well as specialists from UMR5288 lab (Université de Toulouse III; France) and from UMR5292 lab (Centre de recherche en neurosciences de Lyon – CNRS INSERM – Université Lyon I, France).

Inclusion criteria included: being over 18 years of age, having access to the internet, being able to understand the questionnaire instructions, and having been diagnosed with COVID-19, or confirmed by a positive RT-PCR test at any time in 2020, or being suspected of having COVID-19, or having had direct contact with a patient with COVID-19, or having experienced symptoms of any kind during the COVID-19 contingency period. Exclusion criteria were: having a history of allergic rhinitis or a known allergy to odours, being pregnant or lactating women, having a known central neurological disorder, suffering from asthma. In total, 364 individuals were included for the final analysis. In addition, to be included in the final dataset, participants had to respond to the following criteria (Fig. S3): (i) having responded to the full questionnaire, (ii) completing the questionnaire for the first time, (iii) having been diagnosed with COVID-19 and (iv) not having a blocked nose. The characteristics of the participants after the exclusion criteria are presented in Table 1.

#### The questionnaire

The questionnaire was divided in 6 parts. A first section on information related to the COVID, a second including demographic information, a third on their sense of smell (a section of interest in our study), a fourth on their sense of taste, a fifth on their trigeminal sensitivity and other possible disorders (auditory, visual, memory, attentional, affective, mood-related) and a sixth on the chronology of symptoms. In the fifth section of the questionnaire, 4 items were of interest for our analysis: 1/ I have (had) trouble remembering recent activities or events, 2/ I have (had) trouble following a conversation, 3/ I feel more sad or depressed than usual and 4/ Lately I feel worried or more anxious than usual. For the full questionnaire, see Supplementary Note 2. Participants were considered to have reported at least one cognitive difficulty if they answered “Yes” to items 1 (recent memory) and/or 2 (attention to conversation). Similarly, participants were considered to have reported at least one mood disorder if they answered “Yes” to items 3 (sad or depressed) and/or 4 (worried or anxious).

The questionnaire (cronocovid19.org) was completed only once by the participants (some of them provided their email to be contacted for a follow-up), but we only analysed the information at a single time-point. Participants responded at varied times after the onset of the disease (and after the onset of their COVID-19 related OD). As the French survey, it was developed using LimeSurvey.

### Data analysis

Analyses were performed in R 4.0.3 [31] and the level of significance was set at α = 0.01.

For the **French survey**, to examine how factors such as age, gender, OD duration, BMI, smoking status, OD type, OD onset speed, OD constancy impact the effect on cognition, we used hurdle modelling with the “hurdle” function in the R package “pscl” [32]. This statistical approach combines two consecutive steps. First, using a logistic-type regression, the model determines which factors are predictive of the absence of cognitive difficulties. To do this, all values other than 0 are grouped together under the banner “presence of cognitive difficulty” and are encoded by the value 1. Second, using a generalised linear model-like approach with a zero-truncated Poisson distribution, the hurdle model determines the factors predictive of more severe cognitive difficulties (because we explore 6 aspects of cognitive difficulties, the cognitive difficulty can lie on a severity scale ranging from 1 to 6). The explanatory variables were specified independently for each part of the hurdle model. At the beginning of the model selection procedure, the following explanatory variables were introduced: (i) age, (ii) gender, (iii) BMI, (iv) smoking status, (v) OD type, (vi) OD onset speed, (vii) OD constancy, (viii) OD duration and (ix) age by gender interaction. To facilitate comparisons of the estimates, all continuous variables were scaled prior to inclusion in the full model. Then, for each part of the hurdle model, we followed a backward stepwise selection by removing at each step the most non-significant variable.

For the COVID-19 “variant” analysis, due to the spread of the new variants, there was a discrepancy in sample size between the two groups (*n* = 191 for variant-infected and *n* = 54 original-virus-infected participants). To assess whether this discrepancy could lead to a spurious result, we also conducted a propensity score matching procedure. This consisted in a comparison of variant-infected participants in 2021 with participants infected with the original virus in 2020, matched for age, gender, OD type, OD onset speed, OD constancy, OD duration, month of COVID-19 onset, smoking status and BMI, based on their propensity score. The propensity score matching was done with the R package MatchIt [33] and the optimal method. Out of the 2919 participants for which we collected data in 2020, this procedure enabled us to select 191 participants who were as similar as possible to the participants in 2021 infected with new variants. With another χ^2^-test, we compared the proportion of participants infected in 2021 by a variant and who reported at least 1 cognitive difficulty to the proportion of participants infected in 2020 by the original SARS-CoV-2, matched for covariates and who reported at least 1 cognitive difficulty.

The data from the **Mexican survey** consisted of percentages and were analysed by conducting proportion tests with χ^2^ statistics. As we hypothesised that cognitive difficulties would be more frequent in COVID-19 patients with OD than in patients without OD, we used a one-sided χ^2^ in this case.

## Supplementary Information

Below is the link to the electronic supplementary material.

Supplementary Material 1

## Data Availability

The data and code that support the findings of this study are available from the corresponding author upon reasonable request.
