# Supplementary material for: Self-reported cognitive and affective complaints associated with olfactory loss in an online survey of individuals with COVID-19
Source: Eur Arch Otorhinolaryngol. 2025 Oct 18;282(12):6257–67. doi: 10.1007/s00405-025-09660-x (PMC12680891; doi:10.1007/s00405-025-09660-x)
Supplement: Supplementary file 1 — Supplementary Material 1 [file 405_2025_9660_MOESM1_ESM.docx]

**Characterization of cognitive difficulties in individuals with COVID-19-related olfactory loss**

**Supplementary Material**


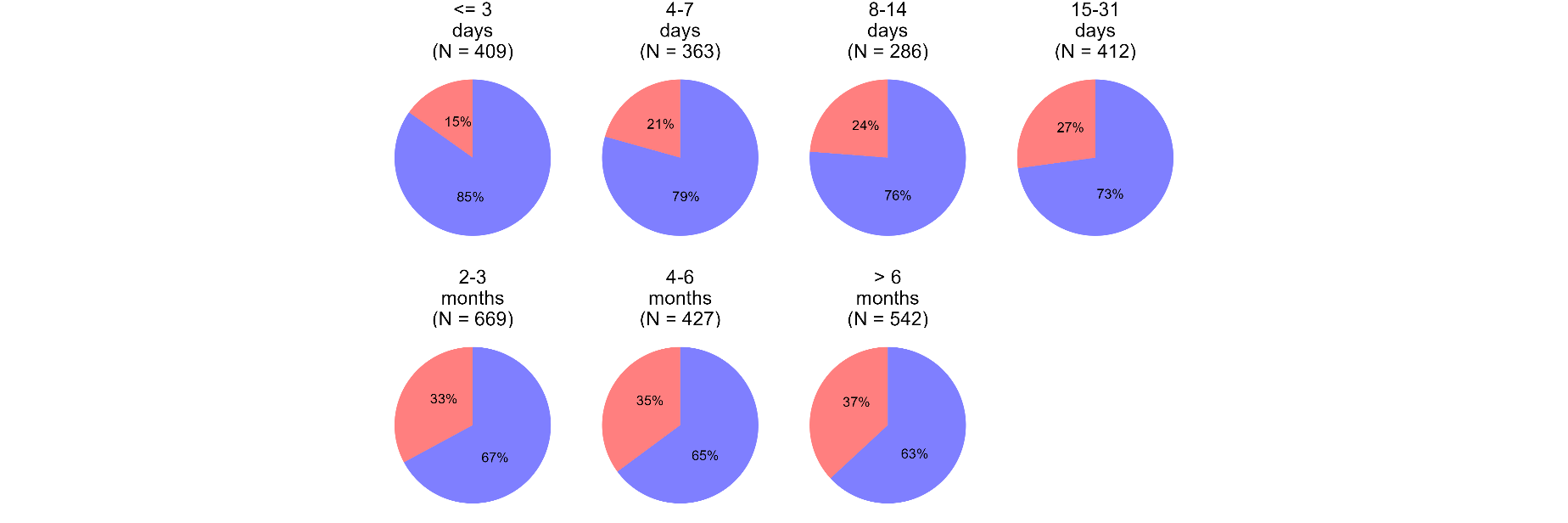


**Figure S1.** Pie charts of the frequency to report at least 1 cognitive difficulty for increasing OD durations. Numbers in brackets indicate the sample size.


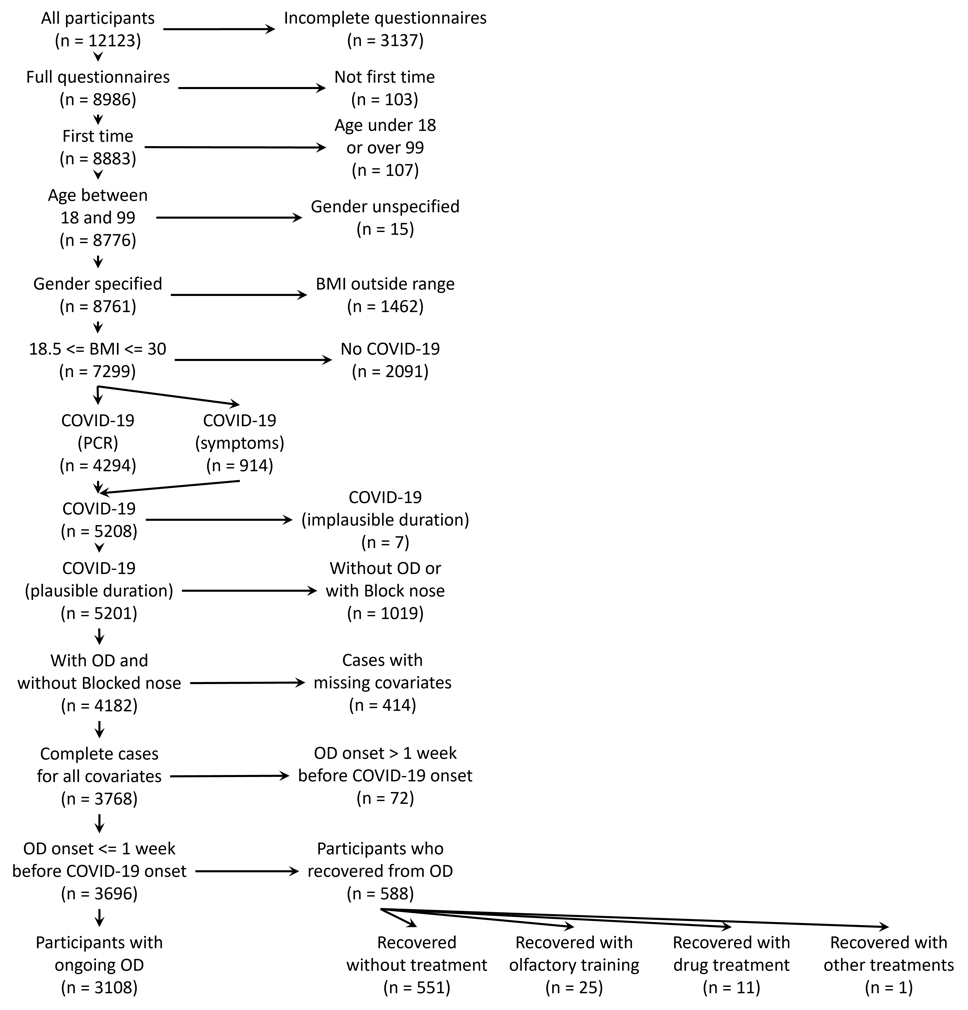


**Figure S2.** Inclusion criteria for the French online survey. Of the 588 people who recovered (see end of decision tree bottom right), 93.7% (n = 551) declared that they recovered without treatment, 4.3% (n = 25) following olfactory training, 1.9% following drug treatment (n = 11) and 0.1% (n = 1) following other types of treatment.


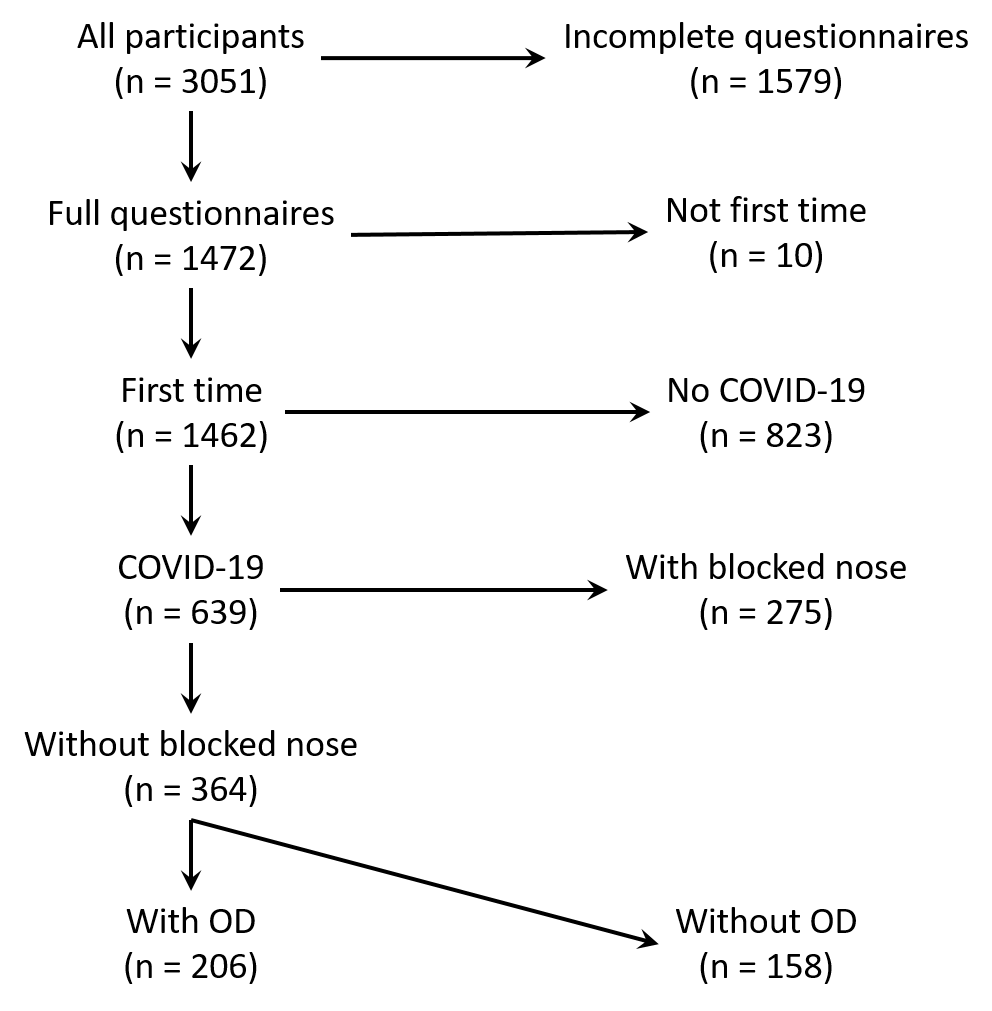


**Figure S3.** Inclusion criteria for the Mexican online survey.

**Supplementary Note 1**

**Quality of life and loss of smell and taste during the COVID-19 health crisis**

This questionnaire was designed by the Neuropop team at the Lyon Neuroscience Research Centre

- - - - - - - - - -

The aim of this questionnaire is to gain a better understanding of the interactions between quality of life and smell and taste disorders in the French population, particularly in relation to the COVID-19 epidemic.

Thank you in advance for taking the time to reply.

Your answers will help us to better understand the possible sensory changes associated with the disease, as well as their consequences on daily life.

Note: this questionnaire takes around 5 to 10 minutes to complete.

Please note that your answers will be taken into account if you answer everything at once: any questionnaire not completed will not be taken into account.

If you have any problems, please contact us at the following address: odorat.info.lyon@gmail.com

If you want to go back, use the "back" button below the questionnaire.

This questionnaire works on Chrome, Edge, Safari, Firefox and Explorer browsers.

.

- - - - - - - - - -

*Note on privacy protection*

*This questionnaire is anonymous.*

*The record of your answers to this questionnaire does not contain any information that could identify you.*

- - - - - - - - - -

Please tick this box if you agree to take part in the survey: 🗆

1. **Preamble**

Is this the first time you have completed this questionnaire?

o Yes

o No

1. **Socio**-**demographic information**
   1. You are:

o Female

o Male

o I do not wish to answer

- 1. How old are you?

___ years old

- 1. How tall are you:

___ cm

- 1. How much do you weigh:

___ kg

- 1. Do you live in France?
- Yes
- No

If yes, please indicate your department of residence: ___ [drop-down list] added end of October 2020

- 1. Are you pregnant?
- Yes, I am pregnant
- I am likely to be pregnant
- No
  1. Do you smoke?
- Yes daily
- Yes occasionally
- No
  1. Do you vape?
- Yes daily
- Yes occasionally
- No

1. **Your sense of smell**
   1. A Over the last few days/weeks, has your perception of smells seemed:

- Normal
- Abnormal
  1. Over the last days/weeks, have you had difficulty perceiving smells (for example, the smell of food, coffee, perfume or soap)?
- Yes
- No

Is this a loss of sense of smell:

- - Slight/partial
  - total

Was the onset:

- - Sudden
  - Progressive

When did the change appear? ___ [date]

Is the problem permanent or do you occasionally experience normal odor perception?

- - Permanent
  - Fluctuating

Do you know the origin of this modification?

- - Yes
  - No

If yes: specify the origin:

- - - Sino-nasal origin (nasal obstruction due to rhinitis, sinusitis, nasal polyp)
    - Traumatic origin (following a shock or accident)
    - Infectious origin (following a viral/bacterial/fungal infection)
    - Congenital origin (from birth)

Has this modificaiton disappeared since?

- - Yes
  - No

If yes: When did it disappear? ____ [date]

How did you recover.

- With a surgery
- With medication, specify the medication(s): ___
- With training
- With other treatments, specify other treatment(s): ___
- No treatment
  1. Over the last few days/weeks, have the smells seemed different from what they usually are ( i.e. they don’t smell the same)?
- Yes
- No
  1. Over the past few days/weeks, have you experienced olfactory hallucinations (phantom smells)?
- Yes: Describe these phantom smells: ___
- No
  1. During these changes in your sense of smell, did your nose feel blocked?
- Yes
- No

1. **Your sense of tase**
   1. Over the past few days/weeks, have you experienced any changes in taste perception (sweet, salty, sour, bitter)?

- Yes
- No

If yes: Specify

- - Difficulty perceiving tastes
  - Tastes seem different from usual
  - I have phantom tastes: Describe which phantom tastes: ___

1. **Your “trigeminal” perceptions**
   1. Over the last few days/weeks, have you experienced any changes in the perception of stinging/ irritation/ cold/ hot (in the nose and mouth) ?

- Yes
- No

If yes: Specify

- - Difficulty perceiving spiciness/irritancy/cold/hotness
  - Spicy//irritant/cold/hot seems different from usual
  - I have phantom sensations of prickliness/irritation/cold/heat: Describe which phantom sensations: ___

1. **About your sensory modifications**
   1. Have you consulted a doctor about your sensory changes?

- Yes
- No

If yes: Who did you consult?

- - A general practitioner
  - A specialist: Please specify which specialty: ___

If yes: What were you told, and if so, what exam/test were you given? ___

If yes: Have you been explicitly diagnosed as dysosmic or dysgeusic (=with disorders of smell or taste)?

- Yes
- No
  1. If you would like to add a comment about your sensory modifications, please write it here: ___

1. **Impact on your daily life**
   1. Are these sensory changes (smell and/ or taste and/or trigeminal sensations) disabling in your personal life?

- Yes
- No

If so : how is it disabling?

- It’s a handicap for my emotional life: Comment: ___
- It is disabling for my social life: Comment: ___
- It is disabling for my professional life: Comment: ___
- It is disabling for other things: Comment: ___
  1. During these sensory changes, did you enjoy eating?
- More than usual
- No more and no less than usual
- Less than usual
  1. During these sensory modifications, did you accidentally let your dishes burn in the oven or on the stove?
- More than usual
- No more and no less than usual
- Less than usual
  1. During these sensory changes, were you wearing perfume?
- More than usual
- No more and no less than usual
- Less than usual
  1. During these sensory modifications, did you add sugar to your dishes to make them more to your liking?
- More than usual
- No more and no less than usual
- Less than usual
  1. During these sensory modifications, did you prefer to eat alone?
- More than usual
- Neither more nor less than usual
- Less than usual
  1. During these sensory modifications, did you have any accidents at home?
- More than usual
- Neither more nor less than usual
- Less than usual
  1. During these sensory modifications, did you add salt to your dishes to make them more to your liking?
- More than usual
- Neither more nor less than usual
- Less than usual
  1. During these sensory modifications, did you add salt to your dishes to make them more to your liking?
- More than usual
- Neither more nor less than usual
- Less than usual
  1. During these sensory modifications, did you smell smoke or gas?
- More than usual
- Neither more nor less than usual
- Less than usual
  1. During these sensory modifications, did you add spicy condiments (chilli, mustard) to your dishes to make them more to your liking?
- More than usual
- Neither more nor less than usual
- Less than usual
  1. During these sensory modifications, did you find pleasure in the scent of flowers?
- More than usual
- Neither more nor less than usual
- Less than usual
  1. During these sensory modifications, have you accidentally eaten spoiled food?
- More than usual
- Neither more nor less than usual
- Less than usual
  1. During these sensory modifications, did you add fatty condiments (mayonnaise, oil) to your dishes to make them more to your liking?
- More than usual
- Neither more nor less than usual
- Less than usual
  1. During these sensory modifications, did you take showers?
- More than usual
- Neither more nor less than usual
- Less than usual
  1. During these sensory modifications, did you burn your clothes when you ironed?
- More than usual
- Neither more nor less than usual
- Less than usual
  1. During these sensory modifications, have you tried to smell your loved ones (children, spouse)?
- More than usual
- Neither more nor less than usual
- Less than usual
  1. During these sensory modifications, if there are other consequences of these odor changes in your daily life, describe them here:
  2. At the time (and/or after) theses changes in smell and /or taste occurred, you also had difficulty to:
- Remember events, dates and appointments
- Remember the location of objects
- Recalling old memories
- Do you remember very recent activities, to the extent that you needed to repeat these actions (for example, checking several times that the door was locked)?
- Follow a program on television or radio
- Follow a conversation intended for you
- Noe of the above

1. **Your current state of health**
   1. Do you usually suffer from respiratory allergies or asthma?

- Yes
- No
- I don’t know
  1. Have you had a broken nose or nose surgery?
- Yes
- No
- I don’t know
  1. Do you have a chronic illness known to affect the sense of smell (e.g. chronic sinusitis, nasal polyp)?
- Yes: Describe your illness: ___
- No
- I don’t know
  1. Do you have a chronic illness?
- Yes: Describe your chronic illness: ___
- No
  1. Are you currently taking any medication?
- Yes: Indicate the medication(s) ___
- No
  1. Have you been tested for Covid-19?
- Yes
- No

If No: Have you tested positive for Covid-19 on the basis of your symptoms alone?

- - Yes^1^
  - No
  - I don’t know

If Yes: How were you tested?

- - By buccal/nasal swab
  - By X-ray
  - By another method (e.g. blood test)
  - I don’t know
  1. Have you been tested positive for Covid-19?
  - Yes^1^
  - No
  - I don’t know

If Yes: Indicate date of onset of illness: ____ [date]

Are you cured today ?

- - - - Yes: Indicate the end date of the illness: ____ [date]
      - No
  1. Have you been sequenced for the so-called "new variants" of coronavirus (British, South African, Japanese...)? *added end of January 2021*
  - Yes
  - No
  - I don’t know

If Yes, have you been identified as a carrier of one of these variants?

- - Yes, which one : _____
  - No

If Yes^1^: Among the symptoms you have experienced, try to determine the order in which they appeared (for those that appeared first, note 1, for those that appeared second note 2, for those that appeared third note 3 etc.).

- Fever: ___
- Cough: ___
- Decreased sense of smell: ___
- Decreased sense of taste: ___
- Reduced sensitivity to spiciness/irritancy/cold/hotness: ___
- Sore throat: ___
- Diarrhea: ___
- Unusual fatigue: ___
- Eating/drinking difficulties: ___
- Shortness of breath: ___

**Thank you for taking part in this survey!**

You can keep up to date with the overall results on the website:

<https://project.crnl.fr/odorat-info/>

**Supplementary Note 2**

COVID-19 ASSOCIATED SYMPTOMS QUESTIONNAIRE

This questionnaire is applied as part of a research study coordinated by the National Institute of Respiratory Diseases "Ismael Cosío Villegas" (INER) to learn more about the symptoms associated with SARS-CoV-2 infection (causing the disease called COVID-19) in the Mexican population, in collaboration with several French institutions.

The questionnaire was designed by an international multidisciplinary team, with the participation of INER (Dr. Santiago Avila, Dr. Mauricio Gonzalez), Neuropop Team at the Lyon Neuroscience Research Center (Dr. Moustafa Bensafi) and the Evolutionary Medicine Team (UMR5288, Dr. D. Pierron/ Dr. Veronica Pereda-Loth) of the University of Toulouse.

- - - - - - - - - -

The purpose of this questionnaire is to better understand the timing of onset of symptoms of COVID-19 disease (diarrhea, loss of taste, cough, fatigue, sore throat, loss of smell, fever, muscle pain, difficulty eating, stomach pain, shortness of breath).

If you have received a positive diagnosis for COVID-19, are a suspect case, have had contact with confirmed cases of COVID-19 or have presented symptoms like those mentioned above during the contingency, your answers will be of great help to better understand the presentation of the disease in the Mexican population and contribute to strengthen epidemiological surveillance.

The questionnaire takes approximately 3 to 5 minutes. Please complete the questionnaire in its entirety, as incomplete questionnaires will not be considered for study analysis.

- - - - - - - - - -

Note on data protection and privacy.

The record of your answers to this questionnaire does not contain any information that identifies you. Participation is COMPLETELY VOLUNTARY and you may withdraw from participation at any time.

- - - - - - - - - -

Check this box if you agree to participate in the survey:

**1. Preamble**

1.1 Is this your first time answering this questionnaire?

- Yes
- No

1.2 Do you have (or have you had) any COVID-19-related symptoms?

- Yes
- No

1.3 Have you ever been diagnosed with suspected COVID-19?

- Yes
- No

If yes:

- Positive result
- Negative result

1.4 The diagnosis for COVID-19 was carried out on what date?

1.5 How was the diagnosis made? with:

- Clinical diagnosis based on symptoms (without laboratory test)
- RT-PCR (nose and throat swab)
- Serological test
- Lung X-ray
- Other method
- Which method?

**2. Sociodemographic information and general health status**

2.1 You are:

- Female
- Male
- I do not wish to answer

2.2 Year of birth:

2.3 What is your height (cm)?

2.4 What is your weight (kg)?

2.5 What is your profession?

2.6 Country where you reside: ___(if Mexico, list state, city and zip code)

(if other than Mexico, what city do you reside in?)

2.7 Do you usually use public transportation?

- Yes
- No

If you answered "Yes", (check all that apply)

- Metro
- Microbus
- Bus
- Combi
- Metrobus
- Train
- Taxi-Uber

2.8 How many people reside in your household?

2.9 How many bedrooms are there in your household?

2.10 Smoking, are you:

- Smoker
- Non-smoker
- Former Smoker

2.11 Are you pregnant?

- Yes
- No
- Maybe
- Don't know
- Not applicable

2.12 Do you have any known central neurological disorder (e.g. Epilepsy, Parkinson's, Alzheimer's...)?

- Yes
- No
- I Don't know

2.13 Do you have any known psychological disorder (e.g., depression, bipolar, schizophrenia, etc.)?

- Yes
- No

2.14 Do you usually have respiratory allergies or Asthma?

- Yes
- No
- I Don't know

2.15 Have you ever had a broken nose or nose job?

- Yes
- No
- I Don't know

2.16 Do you have a chronic condition that affects smell (e.g., chronic sinusitis, nasal polyp)?

- Yes
- No
- I Don't know

2.17 Do you have one (or more) of the following diseases:

- Hypertension
- Heart disease
- Diabetes
- Cancer
- Respiratory disease
- Kidney failure
- Liver disease
- Immune disease

2.18 Are you currently taking any medication?

- Yes
- No

If yes, which one?

2.19 Do you currently take any contraceptive treatment?

- Yes
- No
- I Don't know

If yes, which one?

2.20 At this moment, how do you feel physically (one answer only)?

- Very bad
- Bad
- Good
- Very good

**3. Your sense of smell**

3.1 In general, I have a normal sense of smell:

- Yes
- No

3.2 Lately, I have (or had) difficulty perceiving smells of food, coffee, perfumes:

- Yes
- No

3.3 Lately, I have (or had) a slight loss of smell.

- Yes
- No

3.4 Lately, I have (or had) total loss of smell

- Yes
- No

3.5 Lately, I have (or had) episodes of olfactory hallucinations (phantom smells*)

- Yes
- No

* An olfactory hallucination is not the misinterpretation of an odor, but the perception of an odor when it does not exist. It is discovered most of the time, with the help of people around the patient who do not perceive the same odor.

3.6 On what date did these symptoms appear?

3.7 If these symptoms have disappeared since then, how long did they last?

(in days)

**4. Your sense of taste**

4.1 In general, I have a normal sense of taste:

- Yes
- No

4.2 Lately, I have (or had) difficulty perceiving sweet, salty, sour or bitter tastes: Yes

- Yes
- No

4.3 Lately, I have (or had) a slight loss of taste.

- Yes
- No

4.4 Lately, I have (or had) total loss of taste

- Yes
- No

4.5 Lately, I have (or had) episodes of gustatory hallucinations (phantom tastes*)

- Yes
- No

* A gustatory or gustatory hallucination is not the misinterpretation of an odor, but the perception of an odor when it does not exist. It is discovered most of the time with the help of people around the patient who do not perceive the same taste.

4.6 On what date did these symptoms appear?

4.7 If these symptoms have disappeared since then, how long did they last?

(in days)

**5. Trigeminal perception, auditory, visual, memory, attentional, affective and mood-related changes**

5.1 Lately I have (had) difficulty in perceiving nasal / or oral sensations such as warmth / coolness

- Yes
- No

5.2 Lately I have (had) difficulty hearing (reduced or no hearing in one or both ears / noises in one or both ears).

- Yes
- No

5.3 Lately I have (had) difficulty in seeing (vision changes, blurred vision)

- Yes
- No

5.4 Lately I have (had) trouble remembering

- Yes
- No

5.5 Lately I feel I have trouble following a conversation

- Yes
- No

5.6 Lately I feel sadder or more depressed than usual

- Yes
- No

5.7 Lately I feel worried or more anxious than usual

- Yes
- No

**6. Chronology of symptom onset**

6.1 In what order did the following symptoms appear? Check the appropriate box (First I had these symptoms - These symptoms appeared later - I did not have these symptoms)

- Fever
- Cough
- Decreased Smell
- Decreased Taste
- Sore throat
- Diarrhea
- Unusual fatigue
- Difficulty eating or drinking
- Shortness of breath
- Headache

6.2 Would you agree to be contacted by e-mail if there were additional questions in the framework of the study?

Yes - No

If you answered "Yes", please enter your email address:

THANK YOU VERY MUCH FOR YOUR PARTICIPATION!
